# DNA and RNA Damage, Protection, and Repair in Desiccation-Tolerant Metazoans

**DOI:** 10.3390/biom16070958

**Published:** 2026-06-29

**Authors:** Maria Kamilari, Nadja Møbjerg, Nikos T. Papadopoulos, Antonios Augustinos

**Affiliations:** 1 Department of Plant Protection Patras, Institute of Industrial and Forage Crops, Hellenic Agricultural Organization “DIMITRA”, 26442 Patras, Greece; augustin@elgo.gr; 2 Department of Biology, August Krogh Building, University of Copenhagen, 2100 Copenhagen, Denmark; nmobjerg@bio.ku.dk; 3 Department of Agriculture, Crop Production and Rural Environment, School of Agricultural Sciences, University of Thessaly, 38446 Volos, Greece; nikopap@uth.gr

**Keywords:** desiccation tolerance, oxidative stress, nucleic acid integrity, anhydrobiosis, tardigrades, bdelloid rotifers

## Abstract

Desiccation, ionizing radiation, ultraviolet exposure, and oxidative stress impose severe physicochemical stress that threatens the integrity of both DNA and RNA. Water loss promotes molecular crowding, protein and membrane destabilization, and the accumulation of reactive oxygen species (ROS), while rehydration can intensify oxidative injury and expose lesions accumulated during metabolic suppression. As a result, stress-tolerant metazoans must do more than survive water loss: they must also protect, monitor, and restore nucleic-acid integrity. Here, we review how tardigrades, bdelloid rotifers, *Artemia*, nematodes, and selected insect species preserve genomic and transcriptomic integrity under extreme dehydration, oxidative stress, and radiation-related insults. We compare conserved defence systems, including antioxidant enzymes, trehalose, LEA proteins, heat shock proteins, and core DNA repair pathways. These pathways include base excision repair, nucleotide excision repair, homologous recombination, and non-homologous end joining. We then examine how these conserved mechanisms contrast with lineage-specific innovations, such as the tardigrade proteins Dsup, TDR1, and TRID1, as well as the unusual genome plasticity of bdelloid rotifers. We argue that stress biology of these organisms is best understood through a framework that distinguishes damage prevention during drying from repair and recovery during rehydration. In this framework, extremotolerant metazoans provide biologically informative models for understanding oxidative nucleic-acid damage, redox defence and the molecular logic underlying radioprotection and dry-state preservation.

## 1. Introduction

Maintenance of nucleic-acid integrity is a central requirement for life when challenged with environmental stress. Among the most damaging challenges are desiccation, ionizing radiation, ultraviolet exposure, and oxidative stress, all of which can produce DNA and RNA lesions that compromise replication, transcription, translation, and cellular viability. Reactive oxygen species (ROS), generated both during normal metabolism and under stress, are particularly important because they promote base oxidation, strand breaks, and broader molecular instability. In dehydrating systems, these effects are compounded by the loss of hydration shells, changes in macromolecular packing, and the destabilization of proteins and membranes that normally support nucleic-acid homeostasis [1,2,3,4,5,6,7].

A small but important set of metazoans has evolved the capacity to survive such conditions through cryptobiosis, diapause, or other forms of profound metabolic suppression. In this review, dehydration refers broadly to progressive loss of cellular water, desiccation to severe or near-complete water loss that imposes major physicochemical stress, and rehydration to the return of water and the associated restoration of metabolic activity. We use cryptobiosis in the broad sense of an extreme reversible ametabolic or near-ametabolic state, and anhydrobiosis more specifically for the cryptobiotic condition induced by severe water loss [8]. Tardigrades, bdelloid rotifers, *Artemia* Leach, 1819, certain nematodes, and a limited number of insect systems provide valuable comparative models because they repeatedly face nucleic-acid-threatening conditions associated with dehydration, desiccation, rehydration, oxidative bursts, and, in some cases, radiation exposure. Their survival depends on a layered defence strategy that includes molecular shielding, redox control, chromatin or macromolecular stabilization, and activation of DNA and RNA repair pathways. Across taxa, recurrent protective modules include trehalose, Late Embryogenesis Abundant (LEA) proteins, heat shock proteins, antioxidant enzymes, and intrinsically disordered proteins, whereas repair relies on conserved pathways such as base excision repair (BER), nucleotide excision repair (NER), homologous recombination (HR), mismatch repair (MMR), and, in some lineages, non-homologous end joining (NHEJ) [9,10,11,12,13].

This review focuses on the molecular handling of DNA and RNA damage in desiccation-tolerant metazoans. Rather than treating desiccation tolerance as a primarily ecological or organismal phenomenon, we examine it as a nucleic-acid protection problem. Section 2 outlines the major forms of DNA and RNA injury associated with drying, rehydration, ROS, and irradiation. Section 3 and Section 4 then distinguish damage-limiting protective systems from repair and recovery pathways. Section 5 uses taxon-specific case studies to compare tardigrades, bdelloid rotifers, *Artemia*, nematodes, and selected insects, while Section 6 and Section 7 synthesize comparative principles and discuss translational implications. Particular attention is given to tardigrade-specific proteins such as Dsup, TDR1, and TRID1; the stress-associated genome plasticity of bdelloid rotifers; diapause-associated genome maintenance in *Artemia*; repair regulation in nematodes; and the mechanistic value of insect models, especially *Polypedilum vanderplanki* Hinton, 1951 and desiccation-resistant mosquito eggs [14,15,16,17,18,19,20]. By organizing the review around damage type, protection strategy, and repair logic, we aim to align extremotolerance biology with broader research on oxidative DNA/RNA damage, redox defence, and biomolecular preservation.

## 2. Molecular Consequences of Desiccation for DNA and RNA

Desiccation imposes a profound physicochemical challenge on the cell because the progressive loss of water alters macromolecular crowding, weakens hydration shells, destabilizes membranes and proteins, and changes the intracellular chemical environment in ways that directly and indirectly threaten nucleic-acid integrity [6,7,21]. In this context, DNA and RNA are not merely passive targets of water loss. Rather, they are embedded within a cellular system whose structural and biochemical organization becomes increasingly compromised as dehydration proceeds. The result is a multilevel stress syndrome in which genome stability, transcript integrity, and the ability to resume normal transcriptional and translational activity after rehydration all become vulnerable.

At the DNA level, the major classes of lesions associated with desiccation-related stress include single-strand breaks, double-strand breaks, oxidized bases, abasic sites, and, where ultraviolet exposure is relevant, bulky helix-distorting lesions such as pyrimidine dimers [1,2,4,22,23]. These forms of damage are not unique to desiccation, but dehydration creates conditions that favor their accumulation by increasing oxidative imbalance, disturbing chromatin organization, and impairing the normal buffering capacity of the intracellular milieu [6,7,21]. Oxidative lesions are especially important because reactive oxygen species (ROS) can arise both during the drying phase and during the transition back to metabolic activity upon rehydration. Guanine oxidation, leading, for example, to 8-oxoguanine formation, is particularly relevant because it can alter base pairing and elevate mutational risk if not efficiently repaired [4,24].

The RNA is similarly vulnerable, although its contribution to stress survival has historically received less attention than DNA damage. This imbalance is conceptually problematic. Oxidized, fragmented, or structurally compromised RNA can impair translation, distort post-stress gene expression programs, and delay recovery even when DNA lesions are eventually corrected [5]. Messenger RNAs, ribosomal RNAs, and associated ribonucleoprotein complexes are all sensitive to dehydration-induced physicochemical shifts, particularly when oxidative stress and protein destabilization co-occur. In desiccation-tolerant metazoans, survival therefore depends not only on preserving the genome, but also on maintaining a sufficiently functional transcriptome to support reactivation of cellular pathways during rehydration.

An important mechanistic point is that damage does not accumulate uniformly across the dehydration–rehydration cycle. In many systems, the dry state itself is not necessarily the sole or even the principal source of molecular injury. Rather, rehydration can be a particularly hazardous phase because oxygen influx, mitochondrial reactivation, redox disequilibrium, and restoration of enzymatic activity may together trigger secondary oxidative damage and expose lesions that accumulated during metabolic arrest [6,21]. This distinction is critical for interpreting the molecular biology of desiccation tolerance. Organisms must not only withstand the dry state but also survive the transition out of it without catastrophic loss of genomic and transcriptomic integrity.

For this reason, desiccation tolerance is best framed as a two-stage molecular problem. First, cells must limit nucleic-acid damage during drying and metabolic suppression through antioxidant control, molecular shielding, structural stabilization, and modulation of intracellular chemistry. Second, they must repair and functionally recover from residual damage during rehydration, when replication, transcription, and translation resume [6,21,25]. This framework is especially useful in comparative analyses because it separates mechanisms of damage prevention from mechanisms of damage resolution, two processes that are often conflated in the extremotolerance literature. In the taxa considered in this review, both processes are essential, but different lineages appear to place different relative emphasis on each of them.

**Figure 1:**
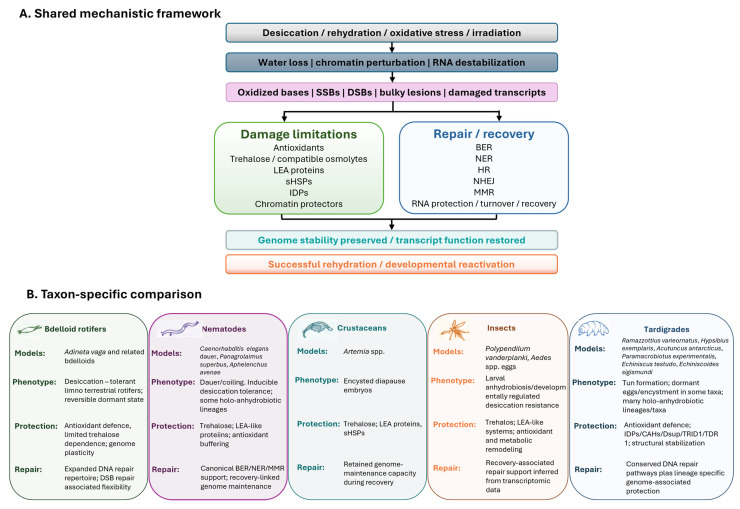
Shared mechanistic framework and taxon-specific comparative overview of nucleic-acid protection and repair in desiccation-tolerant metazoans. (**A**) Desiccation, rehydration, oxidative stress, and irradiation generate physicochemical disturbances that lead to DNA and RNA damage. Survival depends on two coordinated response layers: damage limitation, mediated by antioxidants, compatible solutes, LEA proteins, heat shock proteins, intrinsically disordered proteins, and chromatin protectors; and repair/recovery, mediated by BER, NER, HR, NHEJ, MMR, and RNA protection/turnover pathways. (**B**) Representative taxa are shown in a harmonized comparative format that distinguishes model systems, phenotype, major protective mechanisms, and dominant repair-associated features. This panel highlights both shared molecular logic across lineages and lineage-specific implementations in bdelloid rotifers, nematodes, crustaceans, insects, and tardigrades.

Taken together, these observations place DNA and RNA integrity at the center of desiccation biology. The ability of tardigrades, bdelloid rotifers, *Artemia*, nematodes, and selected insect models to survive severe dehydration cannot be explained solely by dormancy, metabolic suppression, or osmotic adjustment. Instead, their resilience depends on the coordinated protection of nucleic acids before damage accumulates beyond repair, followed by efficient surveillance and restoration as physiological activity resumes [11,25]. This protection-versus-repair framework is summarized in Figure 1.

## 3. Protective Systems That Limit Nucleic-Acid Damage

Survival under extreme dehydration depends not only on the ability to repair damaged nucleic acids after stress exposure, but also on the capacity to reduce lesion formation before damage accumulates beyond recovery. In desiccation-tolerant metazoans, this is achieved through a coordinated set of protective systems that stabilize the intracellular environment, suppress oxidative stress, preserve macromolecular structure, and maintain biomolecular organization in the near absence of water [11,21,26,27]. These systems are especially important because the burden placed on DNA and RNA repair pathways is strongly shaped by the extent to which the cell can buffer structural and chemical instability during dehydration and rehydration. Thus, nucleic-acid maintenance in these organisms should be viewed as the product of two integrated processes: damage prevention and damage resolution.

### 3.1. Antioxidant Defence and Redox Buffering

Among the most widely conserved protective strategies is the control of oxidative stress. The ROS are central drivers of nucleic-acid injury because they promote strand breaks, base oxidation, abasic sites, and broader molecular instability in both DNA and RNA [4,24,28]. Oxidative stress can arise during progressive dehydration, persist in the desiccated state through incomplete redox quenching and residual chemical reactivity, and intensify during rehydration as respiration and enzymatic activity resume [6,21]. For this reason, antioxidant defence is not merely a secondary component of stress tolerance; it is one of the first barriers preventing lesion accumulation across the full dehydration–desiccation–rehydration cycle.

Stress-tolerant metazoans repeatedly show the involvement of enzymatic antioxidant systems such as superoxide dismutase, catalase, and glutathione-related pathways, which act together to constrain ROS before oxidative damage propagates through nucleic acids, lipids, and proteins [28,29,30,31]. In tardigrades, antioxidant responses are consistently associated with survival in desiccated and irradiated states, supporting the view that resistance depends partly on limiting oxidative lesion burden before repair is activated [16,30,32]. Similarly, bdelloid rotifers exhibit exceptional antioxidant protection, which has been proposed as a major contributor to their tolerance of ionizing radiation and recurrent dehydration–rehydration cycles [31,33,34]. In these taxa, oxidative stress control likely reduces the frequency of DNA and RNA lesions that would otherwise overwhelm post-stress recovery systems.

### 3.2. Trehalose and Small-Molecule Stabilization

A second major line of defence involves the accumulation of compatible osmolytes and other small protective solutes, among which trehalose is the best studied in metazoan anhydrobiosis. Trehalose can replace structural water around proteins and membranes, reduce deleterious molecular motion, and contribute to the formation of a stabilized intracellular state during severe dehydration [35,36,37]. Other low-molecular-weight stabilizers, including polyols such as glycerol and certain amino-acid derivatives, may also contribute in some systems, although their comparative role in desiccation-tolerant metazoans is currently less well resolved. This broader stabilizing role is relevant to, e.g., insect systems, where trehalose and other osmoprotectants contribute not only to desiccation-associated survival but more generally to stress tolerance through preservation of macromolecular and membrane integrity, even when the physiological context differs from classical anhydrobiosis [38,39,40,41].

However, trehalose should not be treated as a universal or sufficient explanation for desiccation tolerance. Comparative studies show that its contribution differs markedly across taxa. In *Artemia*, trehalose accumulation is strongly associated with cyst stability and prolonged dormancy under harsh environmental conditions, fitting a model in which carbohydrate-mediated stabilization lowers biomolecular damage during metabolic arrest [42,43,44]. In nematodes, including *Caenorhabditis elegans* (Maupas, 1900) dauer larvae and anhydrobiotic species such as *Panagrolaimus* sp. Fuchs, 1930 and *Aphelenchus avenae* Bastian, 1865, trehalose is a major component of the preconditioning response that supports survival during extreme water loss [45,46,47,48]. In contrast, some tardigrades and bdelloid rotifers show limited or variable dependence on trehalose, indicating that other molecular systems can substitute for or complement its protective role [37,49,50,51,52].

This variability is mechanistically important. It suggests that the unifying principle is not trehalose alone, but the broader use of compatible osmolytes and intracellular stabilizers to preserve organization and reduce oxidative and structural challenges to nucleic acids during drying. This point is particularly relevant because most tardigrades do not appear to synthesize or accumulate substantial trehalose, and bdelloid rotifers likewise show limited reliance on this pathway, with isolated trehalose-related genes in some lineages likely reflecting horizontal acquisition rather than a universal ancestral strategy [37,50,51,52]. Trehalose should therefore be presented as one important protective strategy among several others, rather than as a universal hallmark of desiccation tolerance.

### 3.3. LEA, Heat Shock, and Intrinsically Disordered Proteins

Protein-based molecular shielding is another central component of nucleic-acid protection during dehydration. Late Embryogenesis Abundant (LEA) proteins, heat shock proteins (HSPs), and intrinsically disordered proteins (IDPs) contribute to cellular stabilization by preventing protein aggregation, preserving membrane integrity, and buffering the structural collapse that would otherwise amplify damage to nucleic acids and associated molecular machinery [9,10,53,54]. These proteins do not necessarily interact with DNA or RNA in a single uniform way. Rather, they maintain a stress-compatible intracellular environment in which chromatin, ribonucleoprotein complexes, and repair-associated proteins remain sufficiently organized to support post-stress recovery.

The LEA proteins are especially relevant because they are repeatedly associated with dehydration tolerance across phylogenetically distant taxa. Initially characterized in plants, they are now recognized as important contributors to survival under cellular water deficit in several metazoan systems, including *Artemia*, nematodes, and some tardigrade lineages [9,10,55,56,57]. Their intrinsically disordered nature allows them to remain flexible in hydrated conditions while adopting more protective conformations during dehydration, thereby stabilizing macromolecular assemblies and reducing aggregation or denaturation events that would indirectly compromise DNA- and RNA-associated processes [53]. In tardigrades, however, LEA proteins should not be considered as the sole or dominant molecular basis of desiccation tolerance. Recent synthesis suggests that they are best viewed as one component of a broader protective network that likely acts alongside tardigrade-specific disordered proteins and, in some cases, trehalose-associated stabilization [58].

Heat shock proteins complement this role by acting as molecular chaperones that preserve proteostasis during both dehydration and rehydration. Because nucleic-acid stability depends heavily on the functionality of DNA-binding proteins, chromatin-associated proteins, polymerases, ligases, RNA-binding proteins, and ribosomal components, chaperone-mediated preservation of the proteome is inseparable from the preservation of the genome and transcriptome [59,60]. In *Artemia*, small heat shock proteins are strongly linked to diapause and stress resistance, supporting the view that developmental arrest and molecular chaperoning are tightly coupled [61,62,63]. Similar principles apply in other desiccation-tolerant taxa, even where the exact protein complements differ. Heat shock protein responses appear to be heterogeneous across species and even among members of the same HSP family in tardigrades, suggesting stress-context dependence and possible subfunctionalization rather than a single conserved induction pattern [58].

The IDPs are of particular importance in tardigrades, where they have transformed mechanistic interpretations of anhydrobiosis. Rather than relying exclusively on classical protectants, tardigrades appear to use lineage-specific disordered proteins that stabilize cellular components under extreme stress and help maintain a recoverable intracellular state [12,54,64]. Recent work has further refined this view by showing that the function of disordered proteins depends strongly on the chemical environment and cannot be reduced to a simple universal “glass transition” model [65,66]. This is an important conceptual correction: these proteins are better understood not as static protectants, but as dynamic molecules whose protective capacity emerges from environmentally responsive interactions with the cellular milieu. At the same time, their quantitative contribution to survival, and the extent to which analogous systems operate in other metazoan lineages, remain incompletely resolved. Comparative work also indicates that analogous heat-soluble proteins in heterotardigrades, such as EtAHS, may represent functionally convergent rather than sequence-homologous solutions, reinforcing the view that tardigrade anhydrobiosis relies on a phylogenetically heterogeneous protein toolkit [58].

A further protein class that may be relevant, particularly in tardigrades, is cold shock domain-containing proteins. Comparative transcriptomic analyses have suggested the presence of a tardigrade cold shock protein that may function as an RNA chaperone involved in post-stress translational regulation, especially in the context of freezing-associated stress [16]. At present, however, the evidence is still more suggestive than functionally definitive, and CSPs are therefore best viewed as candidate contributors to RNA stabilization and recovery rather than as established core determinants of desiccation tolerance.

### 3.4. Chromatin-Associated and Lineage-Specific Nucleic-Acid Protection

Some of the most compelling evidence for direct nucleic-acid protection comes from tardigrades, where lineage-specific proteins appear to reduce lesion burden at the level of chromatin itself. The best-known example is Dsup, a DNA-associated protein first identified in *Ramazzottius varieornatus* Bertolani and Kinchin, 1993, which reduces hydroxyl radical-mediated DNA damage and improves radiotolerance when expressed in human cultured cells [14,67]. Dsup is especially important conceptually because it demonstrates that desiccation- and radiation-associated resilience can involve not only generic molecular stabilization, but also a more targeted form of genome shielding.

More recently, Anoud et al. [20] reported TDR1 (Tardigrade DNA Damage Response protein 1), a tardigrade-specific DNA-binding factor induced by ionizing radiation, further supporting the view that tardigrades deploy lineage-restricted mechanisms to maintain genome organization under genotoxic stress. Li et al. [68] also characterized TRID1 (Tardigrade-specific Radiation-Induced Disordered protein 1), a radiation-responsive tardigrade intrinsically disordered protein that appears to promote DNA repair through a phase separation-associated mechanism. Together, these findings argue that tardigrade resilience emerges from a composite system involving redox buffering, molecular stabilization, and direct genome-associated protective factors, rather than from any single protective molecule alone. It would be misleading to imply that all desiccation-tolerant metazoans possess tardigrade-like chromatin protectors, but it would be equally misleading to ignore the fact that such lineage-specific factors can directly reduce lesion burden before canonical repair pathways act. Tardigrades therefore offer not merely another example of stress tolerance, but a model in which direct nucleic-acid protection has become experimentally tractable and mechanistically explicit.

### 3.5. Protection as a Determinant of Repair Burden

Taken together, antioxidant systems, protective solutes, LEA proteins, heat shock proteins, intrinsically disordered proteins, and lineage-specific genome-associated factors all serve a common function: they reduce the number and severity of lesions that must later be resolved by repair pathways. This principle is central to the logic of desiccation tolerance. Repair capacity alone is unlikely to explain survival if the burden of oxidative and structural damage becomes too great during drying or rehydration. Conversely, effective molecular shielding lowers the repair burden and improves the probability that canonical pathways such as BER, NER, HR, and associated recovery mechanisms can restore full nucleic-acid function after stress exposure [25,37].

The protective systems described above are essential for understanding why DNA and RNA repair systems succeed in some desiccation-tolerant organisms and fail in less resilient ones. The following section therefore turns to the conserved and lineage-specific repair pathways that resolve the residual damage that escapes these first lines of defence.

## 4. Core DNA and RNA Repair Pathways

The protective systems described above reduce the burden of molecular injury during dehydration, but they do not eliminate it. Desiccation-tolerant metazoans must also resolve the lesions that escape antioxidant control and molecular shielding, particularly during rehydration, when oxidative imbalance, renewed metabolism, and restoration of macromolecular activity can reveal or amplify pre-existing damage [6,25,26]. Successful survival depends on the integration of protective mechanisms with DNA and RNA maintenance pathways that preserve genome stability and restore functional gene expression after stress. The core repair pathways involved are broadly conserved across metazoans, but their relative importance, pathway composition, and stress-associated deployment differ among lineages.

### 4.1. Base Excision Repair and Oxidative Nucleic-Acid Lesions

Among the major DNA repair systems relevant to desiccation, BER is arguably the most central because oxidative damage is one of the most common molecular consequences of dehydration and rehydration stress. BER is specialized for the removal of small, non-helix-distorting lesions, including oxidized bases, abasic sites, and certain forms of single-strand damage. The pathway typically begins with lesion recognition by a DNA glycosylase, followed by cleavage of the DNA backbone at the resulting abasic site, gap processing, and restoration of the correct sequence by DNA polymerases and ligases [3,23]. Because oxidative lesions such as 8-oxoguanine can arise readily under ROS-generating conditions, BER is especially relevant to stress-tolerant organisms that repeatedly experience redox perturbation [4,24].

In the context of desiccation biology, BER should be viewed as the principal pathway for resolving the oxidative base modifications that persist despite antioxidant defence. This is particularly important because oxidized bases can be mutagenic, interfere with replication and transcription, and compromise recovery if not corrected rapidly after rehydration. Although direct functional studies remain uneven across taxa, the importance of BER is supported by comparative genomics, transcriptomics, and the broader logic of oxidative stress biology in tardigrades, nematodes, *Artemia*, and insect models [16,18,69,70]. Even where the stress-specific activity of individual BER enzymes has not been demonstrated directly, BER remains the most plausible pathway for repairing oxidative base lesions that persist into the recovery phase.

### 4.2. Nucleotide Excision Repair and Helix-Distorting Lesions

Nucleotide excision repair (NER) is the major pathway for removing bulky, helix-distorting lesions, including ultraviolet-induced pyrimidine dimers and certain chemical adducts that interfere with DNA unwinding and polymerase progression [2,22]. In contrast to BER, which targets relatively small modifications, NER excises a short oligonucleotide segment containing the lesion and then restores the missing sequence using the undamaged strand as a template. This pathway is therefore especially important in taxa that experience ultraviolet exposure during dormancy or in environmental settings where desiccation and irradiation co-occur.

The NER broadens the repair framework beyond oxidative lesions, highlighting that desiccation-tolerant organisms often face compound stress regimes rather than dehydration alone. NER has been well characterized in *C. elegans*, making nematodes especially useful for linking canonical metazoan repair biology to stress-resilient survival states. In *C. elegans*, NER includes both global genome repair and transcription-coupled repair, illustrating how cells can protect both silent and actively transcribed genomic regions under DNA-damaging conditions [69]. This duality is conceptually relevant to desiccation tolerance because recovery requires not only genome preservation in the abstract, but also re-establishment of functional transcription after stress.

### 4.3. Homologous Recombination and the Repair of Double-Strand Breaks

Double-strand breaks (DSBs) are among the most severe forms of DNA damage because they threaten chromosome continuity and, if misrepaired, can lead to deletions, rearrangements, or cell death. In stress-tolerant metazoans, DSB repair is therefore a key determinant of whether the genome remains recoverable after dehydration-associated damage or irradiation. Homologous recombination (HR) is the most accurate pathway for repairing DSBs because it uses homologous sequence information—typically from a sister chromatid—as a template for restoration [71]. The HR is particularly relevant in organisms that tolerate significant DNA fragmentation yet resume development or reproduction without obvious catastrophic genome instability.

Bdelloid rotifers are frequently discussed in this context because repeated desiccation and rehydration have been proposed to generate DNA breakage and repair cycles that may influence long-term genome architecture. One longstanding hypothesis is that repeated DSB formation and repair could facilitate the incorporation or retention of foreign DNA through horizontal gene transfer (HGT). However, the interpretation of rotifer genome dynamics should remain careful. The current evidence supports an important role for repair-associated plasticity, but it does not justify simplistic causal claims that desiccation alone fully explains all observed genome architecture or horizontal gene transfer patterns [72,73,74]. This caution is reinforced by the fact that bdelloids share limno-terrestrial habitats with desiccation-tolerant tardigrades, and in some cases nematodes, that experience similar dehydration–rehydration cycles without necessarily showing the same genomic outcomes. A more rigorous formulation is that bdelloid rotifers illustrate how robust DSB repair capacity can coexist with unusual genomic flexibility in a lineage repeatedly exposed to stress-associated molecular disruption.

The HR is also relevant to tardigrades and insect models such as *Polypedilum vanderplanki*, where DNA fragmentation and recovery have been associated with survival under extreme stress [18,75]. In these systems, HR is best interpreted as part of a broader repair-and-recovery program that restores genome integrity after the protective systems described in Section 3 have reduced, but not eliminated, lesion accumulation.

### 4.4. Non-Homologous End Joining and Pathway Heterogeneity Across Taxa

Non-homologous end joining (NHEJ) provides an alternative route for DSB repair by directly rejoining DNA ends without requiring an intact homologous template. Because it is template-independent, NHEJ can operate more rapidly than HR, but at the cost of a greater risk of small insertions, deletions, or junctional errors [71]. In desiccation-tolerant metazoans, NHEJ is therefore potentially valuable during recovery from stress, particularly in non-dividing cells or developmental stages where sister chromatids are unavailable.

However, here we emphasize that stress-associated NHEJ must be handled comparatively and cautiously. Its presence and apparent significance vary across lineages, and canonical pathway architecture may not be fully conserved in all taxa. This issue is particularly relevant in tardigrades, where comparative studies have suggested that some lineages may lack or minimize components of classical NHEJ, implying a greater relative dependence on other repair strategies [12,16]. This is not a minor detail. It means that pathway choice under stress is itself an evolutionary variable and that the logic of DSB repair in extremotolerant animals may not map neatly onto conventional model systems.

### 4.5. Mismatch Repair and Post-Stress Genome Fidelity

Although mismatch repair (MMR) is less frequently emphasized in desiccation studies than BER, NER, or DSB repair, it remains relevant as recovery from stress can involve renewed DNA synthesis and restoration of proliferative activity, especially in developmental stages, embryos, larvae, or tissues that resume active growth after rehydration. The MMR corrects mispaired bases and small insertion–deletion errors that arise during replication, thereby helping to maintain genome fidelity after cells re-enter active growth and division [76]. Its relevance is likely more limited in strictly non-dividing or eutelic adult tissues, and for that reason MMR is best viewed in a desiccation framework as a supporting rather than dominant pathway. The importance of MMR is therefore partly indirect: it does not generally address the primary lesion types generated by dehydration, but it reduces the mutational consequences of recovery-phase replication following stress exposure. Comparative genomic data indicate that core MMR components are broadly conserved among the taxa discussed here, although direct stress-specific functional evidence remains more limited than for BER or DSB repair [16,70]. For this reason, MMR should be viewed as part of the repair landscape potentially supporting the pathways more directly associated with oxidative and fragmentation damage.

### 4.6. The RNA Integrity and Post-Stress Recovery

Upholding RNA integrity likely plays an important, yet under-investigated role, for desiccation tolerant organisms. Oxidative and structural damage to RNA can have immediate functional consequences because modified or fragmented transcripts can impair translation, destabilize stress-response networks, and delay restoration of metabolism after rehydration. Even isolated base lesions in mRNA can reduce translational efficiency or alter decoding outcomes, underscoring the importance of transcript integrity for recovery. Recent perspectives on oxidative RNA damage further support the idea that RNA injury is a meaningful and underappreciated component of cellular stress biology [77].

At the same time, the evidence base for dedicated RNA repair pathways in desiccation-tolerant metazoans is still much less developed than for DNA repair. These organisms likely preserve RNA functionality through a combination of RNA protection, surveillance, selective degradation of damaged transcripts, and re-establishment of transcriptional competence during rehydration, rather than through RNA repair systems sensu stricto [5,78]. The RNA-binding proteins, small heat shock proteins, ribonucleases, and translation-associated quality control mechanisms are all likely contributors to this process, even if their roles have not yet been delineated with the same resolution achieved for DNA repair.

In tardigrades, comparative transcriptomic work has suggested that a cold shock protein may function as an RNA chaperone involved in post-stress regulation of translation, making CSPs a plausible, though still under-validated, component of RNA maintenance and recovery [16]. Hence, RNA maintenance and recovery highlights an important frontier in the field. In other words, current evidence strongly supports the importance of RNA integrity during desiccation survival, but the molecular pathways involved are better described as protective and quality-control systems than as universally defined repair pathways.

### 4.7. Repair Pathways as a Continuum of Post-Stress Recovery

Taken together, BER, NER, HR, NHEJ, MMR, and RNA surveillance-related mechanisms form a continuum of post-stress recovery that complements the protective systems described in Section 3. Their collective function is not simply to remove lesions in isolation, but to restore a cellular state in which replication, transcription, translation, and development can resume without catastrophic loss of informational integrity. This is particularly important in desiccation-tolerant metazoans, where survival often depends on repeated entry into, and exit from, profoundly altered physiological states (Figure 1).

**Figure 2:**
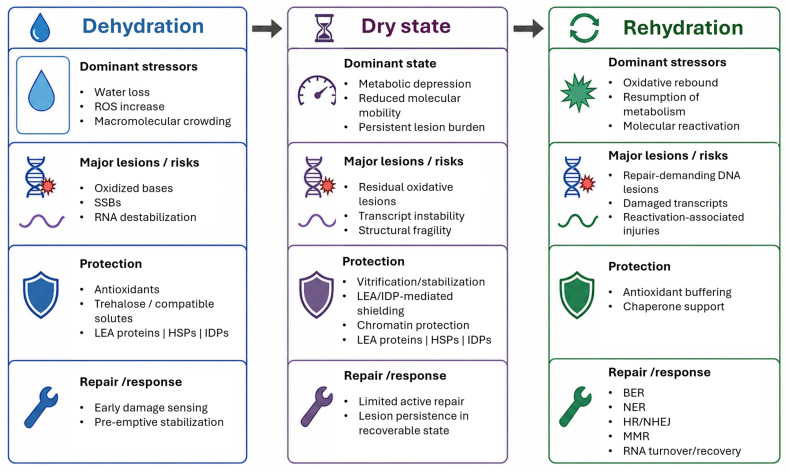
Temporal coordination of protection and repair during dehydration, the dry state, and rehydration. Summary of how dehydration-induced damage, dry-state stabilization, and rehydration-associated repair are temporally coordinated. Survival depends on limiting oxidative and structural lesions during dehydration, preserving a recoverable dry state, and restoring genome and transcript integrity during rehydration.

Yet, repair should not merely be treated as a late, secondary event. It is an integral component of the desiccation-tolerance phenotype. The major comparative question is therefore not whether these organisms possess DNA and RNA maintenance systems—they do—but how different lineages balance protection during drying against repair during rehydration, and which pathway combinations have been most strongly shaped by their ecological and evolutionary histories. The next section addresses this question through taxon-specific case studies. The temporal coordination of lesion formation, protective stabilization, and repair-associated recovery across dehydration, the dry state, and rehydration is summarized in Figure 2. A lesion-centered overview of the principal damage classes and their dominant protective and repair responses is provided in Table 1.

## 5. Comparative Case Studies

### 5.1. Tardigrades: Integrated Nucleic-Acid Protection and Repair Under Extreme Stress

Tardigrades constitute the strongest metazoan model for examining how desiccation tolerance is mechanistically coupled to nucleic-acid protection and repair. Their importance in this context does not arise simply from their broad tolerance of environmental extremes, but from the increasingly detailed evidence that their survival depends on a layered defence system involving antioxidant responses, intrinsically disordered stress proteins, direct chromatin-associated protection, and efficient DNA damage management [12,54,64,75,79,80,81]. This combination makes tardigrades especially informative because they provide one of the clearest examples in Metazoa of how damage limitation and repair are integrated within a single extremotolerant phenotype.

A major advance in understanding tardigrade genome protection came with the identification of the damage suppressor protein (Dsup) in *R. varieornatus* [14]. Hashimoto et. al. [14] showed that expression of Dsup in human cultured cells reduced X-ray-induced DNA damage and improved radiotolerance, thereby demonstrating that a tardigrade-specific factor could directly confer protection against genotoxic stress in a heterologous system. Subsequent work by Chave and colleagues [67] showed that Dsup binds nucleosomes and protects DNA from hydroxyl radicals, providing a mechanistic basis for its protective role. These findings move the discussion beyond generalized stress resilience and identify a direct chromatin-associated mechanism that lowers lesion burden before canonical repair pathways must act.

Recent studies indicate that Dsup is only one component of a broader tardigrade-specific response network. Anoud et. al. [20] identified TDR1, a tardigrade-specific DNA-binding protein induced by ionizing radiation, and proposed that it contributes to genome preservation under genotoxic stress, while Li et. al. [68] described radiation-induced tardigrade-specific intrinsically disordered proteins, including TRID1, and implicated them in DNA damage responses. Clark-Hachtel et. al. [82] further showed that *Hypsibius exemplaris* Gąsiorek, Stec, Morek and Michalczyk, 2018, previously known as *Hypsibius dujardini* [83], strongly upregulates DNA repair pathway genes in response to ionizing radiation, providing direct evidence that tardigrade resilience involves not only lesion prevention but also active mobilization of repair systems. Altogether, these studies suggest that tardigrades do not rely on a single protective factor but instead deploy multiple lineage-specific systems that stabilize chromatin, reduce oxidative lesion formation, and facilitate recovery after irradiation or dehydration-associated stress.

These lineage-specific mechanisms operate within a broader cellular context that includes antioxidant defences and other stress-associated proteins. Comparative transcriptomic work has shown that tardigrades possess diverse repertoires of genes linked to antioxidant activity, DNA repair, and heat-soluble or intrinsically disordered proteins, although the precise composition of these repertoires varies across lineages [16]. This variability is important. It indicates that tardigrade resilience should not be represented as a uniform phylum-wide trait governed by one universal molecular system. Instead, different tardigrade lineages appear to combine conserved repair pathways with distinct lineage-specific protective modules, suggesting that extremotolerance has evolved through multiple related but non-identical molecular solutions [12,16,84].

Tardigrades therefore provide the clearest case in which desiccation and radiation tolerance can be interpreted through the joint action of redox control, molecular shielding, chromatin-associated protection, and damage-responsive repair biology. Their importance in showing that nucleic-acid stability under extreme conditions can be enhanced by both conserved repair pathways and novel taxon-specific proteins, making them central to any comparative analysis of DNA and RNA maintenance in desiccation-tolerant metazoans [14,20,67,68,82].

### 5.2. Bdelloid Rotifers: Repair-Associated Genome Plasticity Under Recurrent Desiccation Stress

Bdelloid rotifers provide a distinct but equally important comparative framework for understanding nucleic-acid maintenance under repeated desiccation stress. Unlike tardigrades, which currently offer the clearest examples of direct chromatin-associated protective factors, bdelloids are especially informative because they combine extreme tolerance of dehydration–rehydration cycles with unusual genome architecture and a diverse repertoire of DNA repair genes [72,73,85]. Their relevance to this review lies in the possibility that recurrent molecular damage has selected for particularly robust repair-associated genome plasticity, rather than in any single iconic protective molecule.

Bdelloids have long been associated with horizontal gene transfer (HGT), and early models proposed that repeated desiccation promotes DNA fragmentation and subsequent genome reassembly, thereby facilitating the incorporation of foreign DNA [85,86]. That interpretation has been shown to require greater caution. Comparative genomic analyses show that both desiccation-tolerant and desiccation-intolerant bdelloid species can share several unusual genomic characteristics, indicating that desiccation alone is unlikely to explain all aspects of bdelloid genome evolution [87]. Wilson and colleagues [74] emphasized that recombination, transfer, and stress in bdelloid genomes remain active areas of debate and that simple one-factor models are insufficient. Accordingly, the most defensible interpretation is not that desiccation directly “causes” HGT, but that recurrent stress has likely imposed strong selection for DNA damage tolerance and genome restoration in a lineage already characterized by unusual genome dynamics.

From a repair perspective, bdelloids are especially compelling because their genomes contain substantial diversity and novelty in DNA repair-related genes. Reference [73] documented a broad and distinctive repair gene repertoire in bdelloid rotifers, supporting the view that these animals possess an expanded or modified DNA maintenance toolkit relative to more conventional metazoan models. This is consistent with a lifestyle in which oxidative stress, DNA fragmentation, and repeated recovery from severe dehydration impose chronic demands on genome stability. In addition, bdelloids show pronounced resistance to oxidative stress and ionizing radiation, further reinforcing the idea that their survival depends on efficient control and repair of nucleic-acid damage [31,33,34,52].

Recent work also indicates that horizontally acquired genes retained in bdelloid genomes can be functionally integrated into adaptive biology rather than existing as passive genomic relics. Although not specific to desiccation, this point matters because it shows that genome plasticity in bdelloids can generate biologically meaningful stress- or defence-related functions [74,87].

Taken together, bdelloid rotifers offer a valuable contrast to tardigrades. Whereas tardigrades highlight direct genome protection through lineage-specific chromatin-associated factors, bdelloids emphasize the long-term evolutionary interplay between repeated stress exposure, robust DNA repair capacity, and unconventional genome dynamics. Specifically, research on bdelloid models (e.g., *Adineta vaga*, *A. ricciae*, and *Philodina roseola*, etc.) Their inclusion strengthens the comparative framework of this review by showing that nucleic-acid stability under desiccation can be achieved not only through direct molecular shielding, but also through repair-oriented genomic adaptability [72,73,74,87].

### 5.3. Artemia: Diapause-Associated Genome Maintenance in Encysted Embryos

*Artemia*’s unique position in desiccation tolerance is due to its best-studied stress-resistant stage being a diapause-stage embryo enclosed in a resilient cyst, not a freely active anhydrobiotic organism. Whereas tardigrades and bdelloid rotifers are often discussed in terms of whole-organism cryptobiosis, *Artemia* illustrates how nucleic-acid protection and repair can be embedded within a developmentally programmed state of prolonged metabolic suppression [88]. Encysted embryos of *Artemia* can persist for extended periods under severe environmental conditions, including high salinity, oxygen limitation, and desiccation, and later resume development when favorable conditions return. *Artemia* is therefore best treated as a model of diapause-associated genome maintenance rather than as a direct analog of tardigrade- and rotifer-style anhydrobiosis. The survival of *Artemia* cysts depends on an extensive protective network that limits molecular damage during dormancy. Trehalose contributes to stabilization of cellular structures during dehydration, while LEA proteins and small heat shock proteins (sHSPs) help preserve protein conformation, membrane integrity, and overall intracellular organization under conditions in which water is severely limited and metabolism is greatly reduced [43,44,56,62,63,89,90]. These protective systems are directly relevant to nucleic-acid maintenance because DNA and RNA stability depend heavily on the preservation of the surrounding molecular environment. If proteins denature, membranes become compromised, or redox homeostasis collapses, nucleic-acid lesions will accumulate more rapidly, and recovery will become less efficient. In this sense, the protective physiology of *Artemia* cysts should be interpreted as a precondition for successful genome preservation during prolonged dormancy.

The physiological interpretation of the *Artemia* cyst state requires some care. Rather than assuming either continuous maintenance activity or absolute biological inactivity, it is safer to frame the cyst as a condition of extreme metabolic depression in which developmental potential and molecular organization are preserved across prolonged arrest [42,91,92]. In this context, heat shock proteins, trehalose, LEA proteins, and related stabilizing systems are relevant not because they imply ongoing active repair throughout dormancy, but because they help explain how cysts remain structurally and functionally recoverable over long periods [62,63,93,94,95]. More broadly, the diapause program appears to preserve a cellular state in which macromolecular integrity is maintained sufficiently well that development can resume once favorable conditions return [44,94].

From the perspective of DNA damage and repair, *Artemia* is best interpreted as a system in which protection and low-level surveillance are likely to operate together. Comparative and genomic studies support the presence of canonical repair pathways relevant to oxidative and structural DNA damage, including components associated with BER, NER, and broader stress-response networks [70]. Because oxidative injury remains a plausible threat during prolonged dormancy and especially during reactivation, BER is likely to be particularly important for resolving base lesions that persist despite antioxidant and chaperone-mediated protection. Likewise, NER may contribute to the removal of bulky lesions accumulated during environmental exposure.

A further strength of the *Artemia* model is that it broadens the conceptual range of stress tolerance without forcing all systems into a single holo-cryptobiont-centered framework [91]. It shows that extensive nucleic-acid preservation can be achieved not only through rapid reversible drying responses, but also through a developmentally programmed form of dormancy that combines biochemical stabilization with selective maintenance of cellular integrity [11,42,44]. *Artemia* should therefore be framed as a model in which diapause-associated stabilization lowers the burden of oxidative and structural nucleic-acid damage, while retained genome-maintenance capacity enables successful recovery when development resumes.

### 5.4. Nematodes: Regulated Cryptobiosis, Oxidative-Stress Control, and Repair-Competent Recovery

Nematodes provide an important bridge between constitutive extremotolerance and regulated stress-induced survival because they include both developmental stages that acquire desiccation resistance conditionally and lineages that tolerate severe dehydration more broadly. *Caenorhabditis elegans* is the most informative model because its dauer larva has become a tractable system for studying how a metazoan can enter a stress-resistant state through coordinated metabolic, biochemical, and gene-regulatory changes rather than through fixed constitutive adaptations alone [17,46]. Anhydrobiotic nematodes such as *Panagrolaimus superbus* Fuchs, 1930 and *Aphelenchus avenae* extend the comparative scope by showing that nematode desiccation tolerance can also involve more extensive genomic and proteomic specialization [45,47].

A major conceptual advantage of nematode literature is that it frames desiccation tolerance as a regulated physiological program rather than as a static trait. In *C. elegans*, dauer larvae survive severe water loss only after appropriate preconditioning, and this survival state is supported by marked biochemical remodeling, including the accumulation of trehalose and the induction of stress-associated protectants [46]. Gade et al. [17] extended this idea by arguing that *C. elegans* possesses a broader cryptobiosis program that can support survival under multiple abiotic stressors, suggesting that desiccation, osmotic challenge, and related stresses are handled through overlapping protective and recovery pathways rather than through entirely separate responses.

From the perspective of nucleic-acid maintenance, nematodes are especially valuable because their desiccation tolerance can be connected directly to canonical DNA repair biology. *Caenorhabditis elegans* has well-characterized pathways for BER, NER, MMR, and DNA damage signaling, making it one of the few systems in which the stress-tolerance literature can be integrated with a substantial broader molecular genetics framework [69,96]. This is particularly important for more general studies focused on oxidative DNA and RNA damage because it allows desiccation biology to be interpreted not simply as a matter of dormancy, but as a problem of preserving genome integrity under conditions that generate oxidative lesions, strand breaks, and broader macromolecular instability. In nematodes too, the key mechanistic question is therefore not whether repair pathways exist, but how their deployment is coordinated with developmental arrest, metabolic suppression, and recovery after rehydration.

Trehalose is especially important in this system because it links biochemical stabilization to survival of the dauer state under extreme dehydration. In *C. elegans*, trehalose accumulation is closely associated with desiccation tolerance and likely reduces the burden of secondary molecular damage by preserving intracellular organization during drying [46]. By stabilizing the cellular environment, trehalose indirectly supports DNA and RNA preservation by reducing the structural collapse and oxidative perturbation that would otherwise increase lesion burden.

Nematode models also help refine the distinction between survival during the dry state and repair-competent recovery after rehydration. Because dauer larvae and anhydrobiotic nematodes resume development only after stress has passed, successful recovery requires that DNA lesions, oxidative modifications, and damaged macromolecules remain within a recoverable range. This makes canonical repair pathways highly relevant even when the exact stress-specific deployment of each pathway has not been fully resolved experimentally. The *C. elegans* literature provides a robust framework for interpreting BER and NER as likely central to recovery from oxidative and structural DNA damage, while apoptosis and checkpoint signaling offer additional means of limiting the persistence of heavily damaged cells [69,96].

Comparative studies in other nematodes further strengthen this interpretation. *Panagrolaimus superbus* has long been used as a model of anhydrobiosis and has yielded molecular evidence for stress-associated pathways, including antioxidants, LEA-like proteins, and other protective systems consistent with desiccation-associated nucleic-acid preservation [45]. More recently, the genome of *A. avenae* has highlighted evolutionary adaptations to desiccation, including features suggestive of extensive molecular specialization for survival in the dry state [47]. These taxa are useful because they show that nematode desiccation biology is not restricted to the inducible dauer program of *C. elegans*. Rather, the phylum contains a gradient of strategies ranging from regulated developmental arrest to more pronounced anhydrobiotic specialization, all of which reinforce the central importance of damage limitation and repair-competent recovery.

As holds for the other models, the issue of upholding RNA integrity in nematodes remains less clearly resolved than DNA repair, but *C. elegans* adds one additional layer of relevance through its well-established RNA interference machinery. Although RNA interference should not be conflated with RNA repair, it does illustrate the sophistication of nematode post-transcriptional regulation and their ability to modulate gene expression rapidly under stress [97,98]. In the context of desiccation tolerance, the safest interpretation is that nematodes likely preserve transcript functionality through a combination of protective molecules, transcript turnover, and tightly regulated recovery of gene expression during rehydration, rather than through uniquely defined RNA repair pathways.

Taken together, nematodes provide a valuable comparative model in which desiccation tolerance can be understood as the product of regulated entry into a stress-resistant state, biochemical protection during dehydration, and repair-competent restoration of function upon rehydration. Their importance to the field lies in linking canonical metazoan DNA repair systems to survival under severe water loss without requiring the unusual lineage-specific proteins that dominate the discussions of selected tardigrade models. In this way, nematodes help demonstrate that robust nucleic-acid maintenance under desiccation can emerge through the strategic redeployment of broadly conserved repair and stress-response pathways [17,46,47].

### 5.5. Insect Models: True Anhydrobiosis in Polypedilum vanderplanki and Desiccation-Resistant Eggs in Aedes

Not all dormancy-associated insect systems contribute equally to a mechanistic understanding of nucleic-acid protection and repair under extreme dehydration. Accordingly, we only include selected insect models in this review. Within Insecta, larvae of the chironomid *Polypedilum vanderplanki* provide the strongest parallel to classical anhydrobiotic metazoans, whereas *Aedes* eggs offer a useful, but conceptually distinct, example of desiccation-resistant developmental stages. Considered together, these systems show that insect resilience to water loss can arise through different physiological routes while still converging on the same central problem: preservation of genome and transcript integrity through dehydration, metabolic suppression, and rehydration.

#### 5.5.1. *Polypedilum vanderplanki*: A Model of True Insect Anhydrobiosis

*Polypedilum vanderplanki*, a chironomid midge native to semi-arid habitats in Africa, remains the clearest insect model of true anhydrobiosis. Its larvae can survive the loss of nearly all body water, enter a metabolically quiescent state, and recover rapidly upon rehydration, a capacity that places this species mechanistically closer to tardigrades and some rotifers than to classical diapause-based insect systems [99,100,101]. This distinction is important for the present review because it means that *P. vanderplanki* can be discussed not simply as an insect with delayed development, but as a system in which desiccation itself is the dominant molecular challenge and in which nucleic-acid preservation must occur under near-complete cellular dehydration.

Several protective systems contribute to this capacity. High levels of trehalose accumulate during dehydration, helping stabilize macromolecules and intracellular organization as water is lost [100]. In parallel, LEA proteins and other stress-associated factors are induced, reinforcing the view that the dry state is actively prepared rather than passively endured [102,103]. Transcriptomic and metabolomic studies further show that this transition is accompanied by major restructuring of carbohydrate metabolism, antioxidant defence, and membrane-associated processes, indicating that the anhydrobiotic state of *P. vanderplanki* is supported by a highly coordinated molecular program rather than by a single protective metabolite or protein [18].

From the perspective of DNA and RNA integrity, *P. vanderplanki* is especially valuable because it appears to couple extensive protective stabilization with a strong post-stress recovery program. Cornette et al. [102] identified numerous anhydrobiosis-related genes in this species, including genes associated with oxidative-stress responses and molecular protection, and heat shock regulatory system has been co-opted as part of the anhydrobiosis program [104]. Although direct functional dissection of individual DNA repair pathways remains less extensively explored than in other model organisms, the available evidence strongly supports a system in which oxidative-stress limitation, molecular stabilization, and repair-compatible recovery are tightly integrated [18].

#### 5.5.2. *Aedes*: Desiccation-Resistant Eggs as a Developmental Model of Nucleic-Acid Preservation

In contrast to *P. vanderplanki*, *Aedes* mosquitoes do not represent whole-organism anhydrobiosis in the same strict sense, but their eggs provide a valuable developmental model of desiccation-associated survival. This distinction should be maintained clearly. The relevance of *Aedes* lies not in complete organismal dehydration, but in the capacity of the egg stage to withstand prolonged dry conditions while preserving viability and developmental potential. In *Aedes albopictus* (Skuse, 1894), egg diapause is well established, and the desiccation resistance of the dormant egg has long been recognized as a major contributor to persistence and invasion success [105,106]. In *Aedes aegypti* (Linnaeus, 1762), although classical diapause is less clear-cut across populations, recent work has shown that temperate populations can display diapause-like responses and produce eggs with delayed hatching and enhanced stress resistance under short-day conditions [19,107].

Mechanistically, *Aedes* eggs are useful because they reveal how desiccation resistance can be developmentally acquired and physiologically regulated. Structural barriers such as the serosal cuticle contribute to water retention, while metabolic remodeling supports persistence under dry conditions [108]. *Aedes aegypti* eggs survive desiccation through rewiring of polyamine and lipid metabolism [109]. Notably, this finding provides direct molecular evidence that desiccation tolerance in mosquitoes depends on active biochemical reprogramming.

Focusing on nucleic-acid stability, *Aedes* eggs should therefore be interpreted as a model of developmentally regulated preservation of viability under water deficit, rather than as a full analog of cryptobiosis. Prolonged survival of embryos in the dry state requires the maintenance of a recoverable cellular condition in which DNA and RNA damage remain within manageable limits. Although the literature currently supports stronger claims for metabolic and structural adaptation than for explicit deployment of defined DNA repair pathways, oxidative-stress buffering and preservation of intracellular organization are clearly central to egg survival [109,110]. In this respect, *Aedes* complements *P. vanderplanki* by extending the insect discussion from true anhydrobiosis to desiccation-resistant dormancy-like developmental stages.

Taken together, these two insect systems broaden the comparative framework used to investigate the mechanisms underlying metazoan desiccation tolerance without weakening its mechanistic focus. *Polypedilum vanderplanki* demonstrates that true insect anhydrobiosis can be achieved through coordinated molecular protection and recovery programs, whereas *Aedes* eggs show that developmentally regulated desiccation resistance can also preserve viability through long dry intervals. Both systems reinforce the central conclusion that survival under severe water loss depends on keeping nucleic-acid damage within a recoverable range and on restoring molecular function efficiently once water becomes available again [18,19,102,104,109].

## 6. Comparative Synthesis: Common Principles and Lineage-Specific Solutions

Despite their substantial phylogenetic distance and contrasting life-history strategies, the metazoan systems reviewed here converge on the common molecular problem: how to preserve nucleic-acid integrity when water loss, oxidative stress, and metabolic interruption destabilize the cell. Tardigrades, bdelloid rotifers, *Artemia*, nematodes, and selected insect models encounter this challenge in different ecological and developmental contexts, yet repeatedly rely on the same broad logic: damage must be limited during dehydration or dormancy, and residual lesions must remain compatible with successful recovery during rehydration or developmental reactivation [11,12,25]. What is shared across these systems is therefore not a single molecule or pathway, but the overall organization of protection and recovery.

A recurring comparative pattern is that survival depends not on repair capacity alone, but on prior reduction in lesion burden. First across taxa, antioxidant defences, trehalose or other protective solutes, LEA proteins, heat shock proteins, and intrinsically disordered proteins reduce the burden of oxidative and structural damage before canonical repair pathways must act [11,56,111]. This is particularly clear in systems such as *Artemia* cysts and *P. vanderplanki*, where molecular stabilization is a prerequisite for survival under prolonged dormancy or near-complete dehydration [18,42,102]. It is equally evident in nematodes, where preconditioning and dauer-associated biochemical remodeling are necessary for severe desiccation tolerance [17,46]. Thus, while repair pathways are essential, they operate most effectively in organisms that first minimize lesion formation through biochemical and structural buffering.

Second, oxidative stress is a central unifying threat, even though the sources and timing of ROS differ among lineages. Whether in tardigrades exposed to irradiation and desiccation, bdelloid rotifers undergoing repeated drying and rehydration, or insect and crustacean dormant stages transitioning back to activity, oxidative injury is repeatedly implicated as a major driver of nucleic-acid damage [31,32,34]. This helps explain why BER and related oxidative-damage responses occupy such a central place in the mechanistic framework of extreme stress tolerance. It also reinforces the point that rehydration is not merely a return to normalcy, but often a high-risk molecular transition in which restored oxygen flux and metabolic activity can amplify residual damage if antioxidant and repair systems are insufficiently coordinated [6,21,25].

Third, canonical repair pathways are broadly conserved, but their deployment is lineage-specific. The BER, NER, HR, and MMR—as well as, in some taxa, NHEJ—are part of the shared metazoan toolkit for maintaining genome stability, yet different groups appear to emphasize different combinations of these pathways according to their ecological demands and molecular innovations [3,16,69,70]. Tardigrades illustrate this clearly. They combine conserved repair pathways with lineage-specific factors such as Dsup, TDR1, and TRID1, thereby supplementing general repair competence with more direct genome-associated protective mechanisms [14,20,67,68]. Bdelloid rotifers, by contrast, are less defined by single iconic proteins and more by the interaction of robust repair capacity with unusual genome plasticity and stress-associated evolutionary innovation [73,74,87]. The *Artemia* model emphasizes diapause-associated stabilization and retained maintenance capacity, whereas nematodes show how canonical repair biology can be embedded within an inducible cryptobiosis program [17,94]. In insects, *P. vanderplanki* represents the strongest example of true anhydrobiosis, while *Aedes* eggs illustrate how developmentally regulated desiccation resistance can preserve viability without fully recapitulating the holo-anhydrobiotic logic of tardigrades or rotifers [18,104,109].

At the same time, these comparisons also expose important limits of universality. Trehalose is highly important in some taxa but clearly not a universal explanation for metazoan desiccation tolerance [37,49,50]. Likewise, direct chromatin-associated genome protection of the type documented for Dsup cannot be generalized across all lineages. Bdelloid HGT, similarly, should not be treated as a generic outcome of desiccation, but as part of a more complicated interplay among stress, genome architecture, and evolutionary history [74,87]. These differences are not peripheral complications; they are central to the current comparative interpretation of the field. They demonstrate that the shared challenge of preserving DNA and RNA under severe stress may have been solved through multiple, only partly overlapping molecular routes.

The RNA constitutes a particularly important area of uncertainty. All the taxa considered here must preserve transcript functionality sufficiently well to support successful recovery, but direct evidence for specialized RNA repair mechanisms remains limited. The current comparative evidence therefore supports a strong shared conclusion on the importance of RNA protection, surveillance, selective turnover, and restoration of transcriptional competence, but a more cautious one regarding dedicated RNA repair pathways sensu stricto [5,78]. This asymmetry should be treated explicitly rather than glossed over, because it points to one of the clearest unresolved questions in the molecular biology of desiccation tolerance.

Taken together, the comparative evidence supports a general model in which stress-tolerant metazoans maintain nucleic-acid integrity through three interacting layers: (i) biochemical and structural stabilization that lowers lesion formation, (ii) antioxidant and molecular buffering that constrain oxidative escalation, and (iii) conserved plus lineage-specific repair systems that restore genome and transcript function during recovery [11,12,25]. This model is preferable to simpler organism- or molecule-centered narratives because it explains both the broad commonalities observed across taxa and the persistent lineage-specific differences that apparently shape how each group survives extreme dehydration.

To distinguish experimentally demonstrated mechanisms from genomic inference and more tentative evidence, the principal comparative features across focal taxa are summarized in Table 2. Tardigrades provide the strongest evidence for direct chromatin-associated protection, bdelloid rotifers for repair-associated genome plasticity, *Artemia* for diapause-associated stabilization, nematodes for regulated cryptobiosis with canonical repair support, and insects for both true anhydrobiosis (*Polypedilum vanderplanki*) and developmentally regulated desiccation resistance (*Aedes* eggs).

## 7. Translational Implications and Biotechnological Applications

The comparative evidence reviewed above indicates that desiccation-tolerant metazoans are valuable not only as models of life under extreme stress, but also as sources of experimentally tractable principles for preserving nucleic-acid integrity under adverse conditions. Their relevance to biomolecular science lies in the fact that many of the same processes that threaten survival during dehydration—oxidative stress, macromolecular destabilization, strand break formation, and loss of transcript functionality—also constrain applications in medicine, biotechnology, agriculture, and biopreservation [12,25,113]. In this respect, extremotolerant metazoans provide a conceptual and molecular framework for designing strategies that either reduce nucleic-acid damage directly or maintain cells and biomolecules in a recovery-competent state.

**Figure 3:**
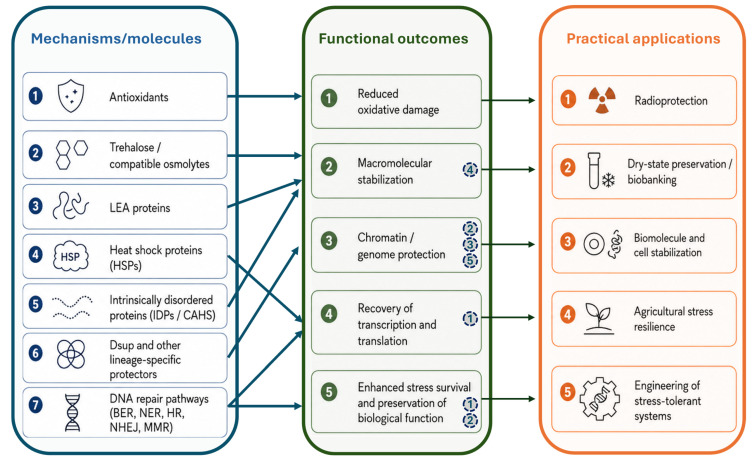
Translational and practical applications of extremotolerance-associated protection and repair systems. Links between protective and repair mechanisms to functional outcomes and potential applications. Solid arrows show primary relationships, while dashed numbered circles indicate secondary contributions to genome protection, recovery, or stress survival.

The major translational implications of extremotolerance-associated protection and repair systems are summarized in Figure 3, linking molecular mechanisms to functional outcomes and potential biomedical, biotechnological, and agricultural applications.

### 7.1. Radioprotection and Genome Shielding

The clearest translational example to emerge from this field is the tardigrade protein Dsup. [14]. In human cultured cells, Dsup expression reduced X-ray-induced DNA damage and improved survival, thereby providing evidence that a protein evolved in an extremotolerant metazoan can enhance genome protection in a heterologous system [14]. Subsequent work demonstrated that Dsup binds nucleosomes and protects DNA from hydroxyl radicals, indicating that its effect is mechanistically tied to chromatin-associated shielding rather than to a nonspecific stress response [67].

More recent work on tardigrade-specific DNA damage-associated factors has further expanded the translational horizon. The identification of TDR1 and TRID1 suggests that selected tardigrades possess a broader repertoire of genome-protective and damage-responsive proteins than previously appreciated, some of which may operate through DNA binding, chromatin stabilization, or phase-separation-related mechanisms that help maintain repair-competent nuclear organization under severe stress [20,68]. Although these newer proteins remain much less developed translationally than Dsup, they strengthen a broader point: extremotolerant metazoans are not merely reservoirs of isolated protective molecules, but potential sources of modular genome-stability strategies that could be adapted for radiobiology, cell preservation, and other applications requiring enhanced resistance to oxidative DNA damage.

### 7.2. Biomolecular Stabilization and Dry-State Preservation

A second major translational area involves the preservation of biomolecules and biologically active materials during drying. The logic here is straightforward. If desiccation-tolerant metazoans maintain a recoverable intracellular state through IDPs, LEA proteins, trehalose, and chaperone systems, then analogous molecules or molecular design principles may be used to stabilize sensitive reagents, enzymes, nucleic acids, and therapeutic formulations outside conventional cold-chain conditions [9,25,66,114]. This is particularly relevant to RNA-containing materials, whose structural fragility and susceptibility to hydrolytic and oxidative damage remain major challenges in biomedical deployment.

In this context, tardigrade and related stress-associated proteins seem especially attractive because their protective capacity does not depend solely on one narrowly defined biochemical reaction, but on their ability to preserve a favorable physicochemical environment for other macromolecules. Intrinsically disordered proteins can limit aggregation, maintain enzymatic activity, and buffer desiccation-associated destabilization in ways that may be useful for preserving biologics during drying and rehydration [54,64,66]. Likewise, LEA proteins and small heat shock proteins provide a conceptual template for designing systems that maintain protein and possibly ribonucleoprotein functionality during storage or transport under fluctuating environmental conditions [53,56]. The key translational principle is therefore not simply “borrowing” one stress protein but reconstructing a protective molecular environment that keeps biomolecules functionally recoverable after dehydration.

### 7.3. Possible Applications in Agriculture and Engineered Stress Tolerance

Molecular mechanisms identified in desiccation-tolerant metazoans also have potential relevance to agricultural biotechnology, particularly in the context of crop resilience to drought and other abiotic stresses. Here, the strongest translational logic lies in the transfer of stress-protective principles, such as improved redox buffering, enhanced molecular stabilization, or reduced DNA lesion burden under oxidative challenge. Although caution is warranted in extrapolating across kingdoms, the fact that Dsup has already been explored in plant systems indicates that tardigrade-derived protection strategies may be adaptable beyond animal cells [115]. More broadly, comparative work on LEA proteins and disordered stress-associated molecules suggests that the functional architecture of desiccation tolerance may be transferrable in modular form, especially where the goal is not full anhydrobiosis, but improved tolerance of dehydration-associated molecular damage [9,25,116].

That said, translational claims in this area should remain disciplined. The existence of a protective molecule in an extremotolerant metazoan does not automatically mean that its function will be reproducible in a plant or industrial setting. Protective effects may depend strongly on expression levels, intracellular context, redox state, and interactions with host proteins or membranes. For this reason, the most defensible translational message is that extremotolerant metazoans offer design principles and candidate molecules, not ready-made universal solutions. We regard this view as both more accurate and more persuasive, especially in a mechanistic review.

### 7.4. Biomedical Relevance and Aging-Associated Genome Instability

The broader biomedical significance of this field lies in its connection to general problems of genome instability. Oxidative DNA damage, declining repair capacity, and impaired stress recovery are central features of aging and are implicated in cancer, neurodegeneration, and other chronic disorders [113]. From this perspective, extremotolerant metazoans are relevant not because humans might ever reproduce full anhydrobiosis, but because these organisms reveal molecular strategies for limiting lesion formation, maintaining repair-compatible cellular states, and restoring function after profound stress. This relevance is further supported by work linking cryptobiosis and stress tolerance to questions of biological aging, including how dormancy, metabolic depression, and recovery may alter the relationship between chronological time and physiological deterioration [117]. Such strategies may inspire new approaches for mitigating oxidative damage in medically important settings, including radiotherapy, cell storage, tissue preservation, and possibly age-associated loss of genomic maintenance.

Recent reviews of tardigrade proteins have emphasized this biomedical potential, particularly for radioprotection and stress resistance in heterologous systems [118]. However, translational enthusiasm should again be matched by caution. The most convincing biomedical applications will be those grounded in specific mechanisms—such as chromatin-associated shielding, redox buffering, or macromolecular stabilization—rather than those relying on broad analogies to “extremophile resilience.” In other words, the translational value of these systems increases as the field shifts from descriptive survival phenotypes to mechanistically resolved stress biology.

### 7.5. From Descriptive Extremotolerance to Predictive Engineering

A final translational priority is methodological standardization. As Marks et al. [25] argue, the field now needs stronger integration across taxa, experimental systems, and molecular scales. This is particularly important for applications in radioprotection, dry preservation, or engineered stress tolerance, where success will depend on distinguishing which traits are broadly conserved, which are lineage-specific, and which require particular biochemical contexts to function effectively. Extremotolerant metazoans have provided experimentally validated leads for radioprotection and biomolecular stabilization, most notably through Dsup and related tardigrade-derived protective frameworks [14,117]. More broadly, they offer design principles for preserving nucleic-acid integrity under stress [12,25,114]. However, the strongest path forward is not broad extrapolation from survival phenotypes, but mechanistic resolution of how protection and repair systems interact at molecular, cellular, and organismal scales.

In this sense, the most important translational contribution of desiccation-tolerant metazoans could be that they force a mechanistic rethinking of what it means to preserve biological information under stress. Their biology shows that resistance is not simply a matter of surviving damage, but of controlling lesion formation, stabilizing vulnerable molecular systems, and coordinating recovery once normal physiology resumes. These are precisely the challenges faced in biomolecular stabilization, genome protection, radiobiology, dry-state biopreservation and engineered stress-tolerant biological systems.

### 7.6. Further Perspectives and Research Directions

A key priority for future research is to move beyond descriptive inventories of stress-associated genes and proteins toward phase-resolved, experimentally validated models of desiccation tolerance. Future studies should distinguish damage-limiting mechanisms during dehydration, stabilizing mechanisms in the dry state, and repair-associated processes after rehydration. This phase-specific framework reflects the organization of anhydrobiotic survival across tardigrades, bdelloid rotifers, *Artemia*, nematodes, and insect models [10,12,42,118]. Integrative approaches combining DNA lesion profiling, RNA integrity assessment, chromatin-state analysis, proteomics, metabolomics, epigenomics, and functional perturbation experiments will be essential for defining causal mechanisms rather than correlative stress signatures, particularly for tardigrade-specific and stress-responsive protective systems such as Dsup, TDR1, TRID1, and intrinsically disordered or heat-soluble proteins [14,20,64,68].

Greater attention should also be given to RNA stability, transcript turnover, ribonucleoprotein organization, and translational reactivation, which remain less well resolved than DNA repair. Current evidence supports the importance of transcript preservation for successful recovery, but is stronger for RNA protection, turnover, and restoration of function than for broadly defined dedicated RNA repair pathways [5,78]. This asymmetry identifies one of the clearest gaps in the literature. Future work should therefore address how oxidative RNA lesions, ribonucleoprotein stability, and post-stress translational recovery contribute to survival under physiologically relevant dehydration–rehydration cycles.

Finally, broader and more standardized comparisons across tardigrades, bdelloid rotifers, *Artemia*, nematodes, and desiccation-tolerant insect stages are needed to separate conserved principles of nucleic-acid preservation from lineage-specific innovations. Comparative genomic and transcriptomic studies have already shown that extremotolerant metazoans use both shared stress-response systems and taxon-specific molecular solutions, but differences in experimental design, stress intensity, developmental stage, and recovery endpoints still limit direct synthesis across lineages [16,54,72,102,119,120]. In parallel, organism-level stress tolerance should be translated into mechanistically testable cellular models to determine which protective and repair-associated mechanisms are transferable, context-dependent, or effective only in specific molecular combinations. Such comparative and mechanistic work could provide the foundation for a more predictive understanding of how metazoan cells maintain genome and transcript function under extreme water loss.

## 8. Conclusions

Desiccation-tolerant metazoans demonstrate that survival under extreme water loss depends on maintaining nucleic-acid damage within a recoverable range. Across tardigrades, bdelloid rotifers, *Artemia*, nematodes, and desiccation-tolerant insect stages, this is achieved through the coordinated action of oxidative-stress control, molecular stabilization, chromatin and genome protection, and repair-associated recovery after rehydration. Rather than preventing all damage, these systems preserve the structural and functional conditions required for genome integrity, transcript recovery, and cellular reactivation.

Collectively, the evidence reviewed here indicates that desiccation tolerance is not governed by a single universal molecule or pathway, or by one lineage-specific innovation alone. Instead, different metazoan lineages have evolved partially overlapping molecular solutions to the shared challenge of preserving biological information during dehydration, the dry state, and rehydration. This convergence in broad functional outcome does not necessarily imply mechanistic identity. Tardigrades exemplify the role of lineage-specific proteins within broader protective and repair-associated networks; bdelloid rotifers highlight repair-associated genome plasticity and unusual DNA maintenance repertoires; *Artemia* illustrates diapause-associated preservation of nucleic-acid integrity; nematodes show how canonical repair systems can be incorporated into inducible cryptobiotic programs; and insect models demonstrate that nucleic-acid preservation under water loss can arise through either true anhydrobiosis or developmentally regulated resistant stages. Together, these systems establish desiccation-tolerant animals as valuable comparative models for understanding how DNA and RNA integrity can be protected, repaired, and functionally restored under otherwise highly destabilizing conditions.

## Figures and Tables

**Table 1:** Lesion-centered overview of nucleic-acid damage and dominant protective/repair responses under desiccation-associated stress. This table summarizes the principal lesion classes relevant to dehydration–rehydration stress, their major physicochemical triggers, the protective systems that limit lesion burden, and the dominant repair or recovery pathways most likely to restore function.

Lesion/Molecular Problem	Principal Triggers Under Desiccation-Associated Stress	Major Protective Systems Limiting Lesion Burden	Dominant Repair/Recovery Pathways	Representative Taxa/Strongest Examples
**Oxidized bases**	ROS accumulation during dehydration and especially rehydration	Antioxidants, trehalose-associated stabilization, IDPs, HSPs	BER	Tardigrades, bdelloid rotifers, nematodes, *Artemia*
**Single-strand breaks (SSBs)**	Oxidative stress, strand cleavage during stress transitions	Antioxidant buffering, chromatin stabilization, proteostasis maintenance	BER; ligation-associated recovery	Tardigrades, nematodes
**Double-strand breaks (DSBs)**	Severe oxidative stress, irradiation, chromatin disruption, fragmentation during drying/rehydration	Chromatin protectors, redox control, intracellular stabilization	HR; variable NHEJ	Tardigrades, bdelloid rotifers, *P. vanderplanki*
**Bulky helix-distorting lesions**	UV exposure, irradiation-associated lesions, and some chemical stresses	General molecular shielding and dormancy-associated stabilization	NER	Especially well supported in nematodes; inferred more broadly
**Damaged/oxidized transcripts**	ROS, physicochemical destabilization, rehydration-associated stress	RNA-binding proteins, HSPs, IDPs, maintenance of ribonucleoprotein integrity	RNA protection, surveillance, selective turnover, and recovery of transcription/translation	Evidence across taxa remains limited/indirect
**Global chromatin/genome instability**	Water loss, molecular crowding, ROS, and repeated stress cycles	Direct chromatin-associated protectors, IDPs, antioxidant defence	Coordinated repair plus recovery-phase genome maintenance	Tardigrades (Dsup, TDR1, TRID1) and bdelloids
**Loss of recovery competence after dormancy**	Prolonged arrest, oxidative drift, and macromolecular destabilization	Trehalose, LEA proteins, sHSPs, retained maintenance capacity	Integrated repair-compatible reactivation	*Artemia*, nematodes, and insect dormant stages

**Table 2:** Summary of comparative evidence for nucleic-acid protection and repair in selected desiccation-tolerant metazoans. Entries are intended to reflect the evidentiary basis of each feature rather than simple presence or absence. **Direct evidence** indicates experimental demonstration in the focal taxon under relevant stress conditions. **Genomic/transcriptomic evidence** indicates inference from gene repertoire and/or stress-responsive expression data. **Limited/indirect evidence** indicates plausible support without full mechanistic resolution. **Not established** indicates insufficient evidence at present.

Molecular Feature	Tardigrades	Bdelloid Rotifers	*Artemia*	Nematodes	Insects (*P. vanderplanki*/*Aedes* Eggs)
**Oxidative-stress buffering**	Direct evidence [30,32] genomic/transcriptomic support [16]	Direct evidence [31,34,52]	Direct evidence [43,44]	Direct evidence [8,17,46,47]	Direct evidence [18,109]
**Trehalose-associated stabilization**	Variable/lineage-dependent [37,50,51]	Limited to moderate; lineage-dependent [37,49,52]	Direct evidence [42,43,44]	Direct evidence [45,46,47]	Direct evidence [18,100,109]
**LEA proteins/LEA-like protection**	Genomic/transcriptomic evidence; lineage-dependent [12,14,16,54]	Limited/indirect evidence [52,87]	Direct evidence [44,56]	Direct evidence [45,47]	Direct evidence in *P. vanderplanki* [102,103]; not established in *Aedes* eggs
**Heat shock proteins/chaperone systems**	Direct evidence [54,64,65,66,68]	Direct evidence [34,52]	Direct evidence [63,93,94]	Direct evidence [17,46]	Direct evidence (*P. vanderplanki*: [104]; *Aedes*: [19])
**Intrinsically disordered proteins (IDPs)**	Direct evidence [54,64,65,66,68]	Limited/indirect evidence [74,87]	Limited/indirect evidence [56,70]	Limited/indirect evidence [45,47]	Limited/indirect evidence; stronger in *P. vanderplanki* [18,102] than *Aedes*
**Direct chromatin-associated protection**	Direct evidence [14,20,67,68]	Not established	Not established	Not established	Not established
**BER-associated recovery**	Genomic/transcriptomic evidence; likely important [12,16,82]	Genomic/transcriptomic evidence [73,74]	Genomic/transcriptomic evidence [70]	Direct evidence/strong model support [69,96]	Genomic/transcriptomic evidence (*P. vanderplanki*: [18]; *Aedes*: indirect)
**NER-associated recovery**	Genomic/transcriptomic evidence [12,16,82]	Genomic/transcriptomic evidence [73]	Genomic/transcriptomic evidence [70]	Direct evidence/strong model support [69]	Limited/indirect to genomic evidence [18,19]
**HR-associated DSB repair**	Genomic/transcriptomic evidence [16,75,82]	Strong indirect/genomic evidence [73,74,85]	Genomic/transcriptomic evidence [70]	Genomic/transcriptomic evidence [69,96]	Limited/indirect evidence (*P. vanderplanki*) [18]
**NHEJ-associated DSB repair**	Variable/possibly reduced in some lineages [12,16]	Genomic/transcriptomic evidence [73]	Limited/indirect evidence [70]	Genomic/transcriptomic evidence [93]	Limited/indirect evidence
**RNA protection/turnover/recovery**	Limited/indirect evidence [16,54,64,77]	Limited/indirect evidence [77,87]	Limited/indirect evidence [44,77,94]	Limited/indirect evidence [17,77]	Limited/indirect evidence [19,77,109]
**Lineage-specific innovations**	Dsup, TDR1, TRID1, CAHS/heat-soluble proteins [14,20,68,112]	Repair-associated genome plasticity; HGT-linked novelty [74,85,87]	Encysted diapause embryo; cyst-associated stabilization [42,70]	Dauer-associated cryptobiosis program [17,46]	True anhydrobiosis in *P. vanderplanki*; developmental desiccation resistance in *Aedes* eggs [18,19,109]

## Data Availability

No new data was created in this study.
